# Interspecific Competition Influences Fitness Benefits of Assortative Mating for Territorial Aggression in Eastern Bluebirds (*Sialia sialis*)

**DOI:** 10.1371/journal.pone.0088668

**Published:** 2014-02-06

**Authors:** Morgan R. Harris, Lynn Siefferman

**Affiliations:** Appalachian State University Biology Department, Boone, North Carolina, United States of America; University of Melbourne, Australia

## Abstract

Territorial aggression influences fitness and, in monogamous pairs, the behavior of both individuals could impact reproductive success. Moreover, territorial aggression is particularly important in the context of interspecific competition. Tree swallows and eastern bluebirds are highly aggressive, secondary cavity-nesting birds that compete for limited nesting sites. We studied eastern bluebirds at a field site in the southern Appalachian Mountains that has been recently colonized (<40 yr) by tree swallows undergoing a natural range expansion. The field site is composed of distinct areas where bluebirds compete regularly with tree swallows and areas where there is little interaction between the two species. Once birds had settled, we measured how interspecific competition affects the relationship between assortative mating (paired individuals that behave similarly) and reproductive success in eastern bluebirds. We found a strong tendency toward assortative mating throughout the field site. In areas of high interspecific competition, pairs that behaved the most similarly and displayed either extremely aggressive or extremely non-aggressive phenotypes experienced higher reproductive success. Our data suggest that interspecific competition with tree swallows may select for bluebirds that express similar behavior to that of their mate. Furthermore, animal personality may be an important factor influencing the outcome of interactions between native and aggressive, invasive species.

## Introduction

Quantifying consistent individual differences in behavior across different spatial and temporal contexts [1], [2] may be important to understanding how ecological and evolutionary forces shape populations, communities, and ecosystems [3]. To explore ecological processes, the contribution of individuals to the overall function of populations within an integrated ecosystem must be considered [4]. Individual behavior dictates how individuals interact with their environment, and in turn, affects how other individuals or species respond to their environment. Moreover, interspecific competition has implications for community and population structure [5], character displacement [5], settlement patterns [6], and invasiveness or susceptibility to invasions [7], while individual behavior influences reproductive output and survivorship [8]–[12].

Boldness to predators, exploratory behavior, and territorial aggression, are a few traits used to quantify individual differences in behavior. However, the “shy-bold continuum” [1], [13] and exploratory behavior [9], [14]–[18] are well studied, while territorial aggression as a repeatable trait is discussed less often in the literature [but see 19–23]. Indeed, aggression – defined as behavior directed toward individuals that are intended to or have the capacity to harm or intimidate an individual [24] – is ecologically important due to its inherent risk of injury [21] and/or death [10]. Yet the implications of aggression for reproductive success can vary with species, local environment, and life-history tradeoffs [22], [25]–[29]. Few studies have examined relationships between interspecific competition and the expression of consistent individual behavior. One exception is Webster et al.’s [5] study of two species of sticklebacks (*Gasterosteus sp.*), which demonstrated that individual boldness affects the outcome of interspecific competition for resources. Instead, most research has focused on the effects of individual behavior on the outcome of intraspecific contests. For example, Rosvall [20] and Cain & Ketterson [30] found that more aggressive individuals are more competitive and have higher reproductive success.

Monogamous birds generally defend breeding territories and aggression is an important component to territorial defense. For obligate secondary cavity-nesting birds (i.e. those that do not excavate their own nesting cavities), nest sites are limited [31], especially in human-altered landscapes [32]. As a consequence of competition for nesting cavities, both males and females of many secondary cavity-nesters are extremely aggressive [33]–[35]. Moreover, biparental care is the norm and parents often experience tradeoffs between territorial aggression and parental care. Individuals that devote a great deal of time and energy to parental effort may do so at the cost of territory and nest defense [19], [36], and thus may allow intruders to usurp nesting cavities [37]. Yet, extremely aggressive nest defense behavior can lead to insufficient parental care [19]. It stands to reason that, in species with biparental care of young, the behavior of both parents can influence reproductive success [reviewed in 38]. When choosing mates, individuals may mate assortatively or disassortatively for behavioral traits. In disassortative pairs, members may exhibit a sort of division-of-labor that leads to increased performance or rear young that exhibit an intermediate behavioral phenotype that leads to high survivorship [8]. In assortative pairs, members may cooperate more efficiently and thus experience mutual reproductive benefits [38]. For example, great tit (*Parus major*) pairs that display similar and extreme exploratory behavior produce the highest-quality offspring [9]. Currently, the definition of assortative mating is unclear. The term assortative mating implies that paired mates choose their partners based on particular traits [39]. However, because researchers rarely measure behavioral traits prior to pairing, assortative mating is often defined as similar behavior among mated pairs [9], [12]. Here, to simplify wording, we refer to assortative mating as the pattern of mated pairs in a population [39]–[41].

Eastern bluebirds (*Sialia sialis*) are a secondary cavity-nesting species that exhibits repeatable aggressive behaviors in the face of simulated territorial intrusions (STIs) and there is a large amount of variation in territorial aggression within populations [23]. Eastern bluebirds have a wide geographic range that covers nearly all of the eastern United States from central Ontario south to central Texas [33]. Throughout this range, bluebird populations face very different environmental constraints and challenges. For example, bluebirds that breed in northern regions compete with tree swallows (*Tachycineta bicolor*) for nest cavities while bluebirds breeding in more southern locations do not [33], [34]. Indeed our field site is a mosaic of distinct areas where bluebirds compete with tree swallows for nesting cavities and areas with little interaction between the two species. Eastern bluebirds and tree swallows act aggressively toward one another [33], [34], [37] and the result of competition between the two species is often eviction of the bluebird pair from the nest-box [37, Harris, pers. obs.]. In this system it seems that as the breeding season continues, interspecific competition from tree swallows has a larger effect on breeding bluebirds than does intraspecific competition. Intraspecific competition certainly has a large effect on settlement in bluebirds [42], but once bluebirds have established nesting territories aggressive interactions are rare [Harris pers. obs.]. For example, at our field site once bluebirds have begun nesting, we have not documented nest usurpation by other bluebirds, but 15% of bluebird nests were usurped by tree swallows. Swallows occur in high densities, are aerial foragers that generally forage within 300 m of their nest, unmated ‘floaters’ are common and this species readily mobs other species [34].

Here we examine whether interspecific competition with tree swallows influences the relationship between territorial aggression and reproductive success in eastern bluebirds. First, we explore whether individuals within bluebird pairs are mated assortatively for territorial aggression (mated males and females demonstrate similar responses to simulated territorial intrusions). Second, we quantify how interspecific competition influences the relationship between assortative mating for territorial aggression and reproductive success.

## Methods

### Ethics Statement

This study was carried out in strict accordance with the recommendations in the guide for the Care and Use of Animals for Research, Teaching, or Demonstration provided by Appalachian State University through the Institutional Animal Care and Use Committee (IACUC). The methods were approved by IACUC at Appalachian State University (permit number: 12-09). All animals were handled in such a way to reduce stress and avoid physical harm. Research was conducted under North Carolina State and U.S. Fish and Wildlife permits. All adults were released in their home territory and nestlings returned to their nest-boxes. We had permission from all landowners.

### General Field Methods

We studied eastern bluebirds breeding in Watauga County, NC during the 2012 breeding season. We monitored egg laying, hatching, and fledging success of eastern bluebirds and tree swallows. In all bluebird nests, we measured mass (±0.1g) of nestlings at age 14 days (hatch day = day 1). From the time they hatch until they are about 11 days old, nestlings increase rapidly in mass, but by age 13 days, the mass of nestlings begins to asymptote [43] and nestlings fledge between age 15 and 21 days [33]. Hence, the mass of nestlings 14 days after hatching is an accurate estimate of fledgling mass. Nestling mass is an important measure of reproductive success in birds because, in many species, nestling condition is positively related to the probability of becoming a recruit in the following breeding season [44]. Therefore, we used the number of fledglings and the mass of nestlings at age 14 days as two proxies of reproductive success. We captured breeding bluebirds and fitted them with an aluminum, numbered USGS band and three plastic colored leg bands to facilitate subsequent identification.

### Using Past Data to Assess Habitat Quality and Predict Interspecific Competition

The field site included five distinct spatial clusters of nest-boxes, hereafter referred to as ‘zones’. We defined zones as areas where nest-boxes were <0.50 km apart (mean = 0.15 km) while zones were >1 km apart (mean = 1.32 km). We created a map of the field site in Google Earth [45] and placed 300 m radius buffers around each bluebird nest to calculate the local density of tree swallow nests within each buffer during the 2012 breeding season. From this, we calculated the mean density of tree swallow nests per zone [46]. Tree swallows normally forage within 300 m of their nest-box so a 300 m radius buffer from a bluebird nest should encompass the area where interspecific interactions are likely to occur [47].

### Aggression Trials

We conducted simulated territorial intrusions (STIs) to measure territorial defense aggression at each eastern bluebird nest (n = 63). STIs were conducted during late incubation (day 10–14) for all birds and again during nestling rearing for a subset of parents (n = 17 pairs). We used live caged male and female bluebirds as stimulus models due to their availability at the beginning of the field season. Western bluebirds do not differ in their reaction to bluebirds or tree swallows, so we feel the use of a conspecific model represents a comparable territorial intrusion for a general measure of territorial aggression [19]. The models were captured >30 km from the field site. We simultaneously placed one male and one female captive bluebird in separate cages 1 m from the focal pair’s nest-box and broadcasted bluebird vocalizations (‘chatter’). We quantified aggressive behavior separately for male and female bluebirds. Before beginning the trial we visually searched the territory to confirm that the breeding pair was in the area. Once a focal bird responded (male or female chattered or moved toward the intruders), we observed behavior for 10 minutes. Although bluebirds rarely dove or physically attacked the model, most landed on the intruder’s cage. We calculated aggression as the latency time (seconds) from the start of the trial until each focal bird landed on the cage of the same-sex conspecific intruder. The time it takes an individual to respond to an STI likely has ecological importance so the total time from the start of the trial until landing on the cage was used. The longest trial conducted lasted 23 minutes because it took the focal pair 13 minutes to respond to the intrusion. Thus, if a bird did not land within 10 minutes after responding, they were given a score of 1400 seconds.

### Statistical Methods

All statistical tests were performed using SPSS v.20 statistics software [48]. For the subset of birds that experienced STIs twice, we examined repeatability of aggressive response using intraclass correlations [49]. We also used intraclass correlations to determine whether mated pairs behaved similarly.

To elucidate differences in the amount of interspecific competition between the zones we used a univariate analysis of variance (ANOVA) with tree swallow density as the dependent variable and zone as the fixed factor. To test the effect of parental behavior and of interspecific competition on nestling quality and reproductive output, we used two general linear mixed models (GLMM). In each model, nest ID was the random factor, male and female behavior were covariates, and the level of interspecific competition (high and low) was the fixed factor. Furthermore, because nestling sex, brood size and hatch date could influence reproductive output, these variables were also included in the original models. We used a stepwise backward procedure for simplification of the mixed models and tested interactions between the fixed factors and covariates. We also used a GLMM to investigate the difference in nestling mass between high and low competition sites. Also, to determine the effect of parental behavior on reproductive output we used a univariate analysis of covariance (ANCOVA). The ANCOVA included number of nestlings fledged as the dependent variable, male and female behavior as the covariates, and hatch date and competition level as fixed factors.

## Results

### Distribution of Interspecific Competition

The average (± SD) tree swallow densities, measured in nests/territory (n/t –300 m radius buffer) were as follows: zone 1 = 4.16 n/t ±1.74; zone 2 = 1.72 n/t ±0.88; zone 3 = 1.25 n/t ±0.97; zone 4 = 6.71 n/t ±1.51; zone 5 = 4.00 n/t ±2.00. The overall ANOVA revealed a significant effect of zone on tree swallow density (*df* = 4, *F* = 29.46, *p*<0.001) and Fisher’s LSD post-hoc tests revealed that zones 2 and 3 had significantly lower tree swallow densities compared to zones 1, 4, and 5 (all *p*≤0.001). Zone 4 had significantly higher density than any of the other 4 zones (all *p*<0.001), but we categorized zones 1, 4, and 5 together as ‘high competition’ sites because densities were all significantly higher than zones 2 and 3. Therefore, zones 2 and 3 were categorized as ‘low competition’ sites.

### Repeatability

Female eastern bluebirds exhibited significantly repeatable aggression (*df* = 15, *intraclass correlation = *0.69, *p* = 0.02). However, male aggression was not significantly repeatable (*df* = 16, *intraclass correlation = *0.159, *p* = 0.37).

### Assortative Mating for Territorial Aggression

There was a significant positive relationship between the aggression of paired males and females (*df* = 62, *intraclass correlation* = 0.69, *p*<0.001). Moreover, individuals within a pair behaved similarly (assortative mating) in both low (*df* = 25, *intraclass correlation* = 0.69, *p*<0.001) and high competition sites (*df* = 22, *intraclass correlation* = 0.54, *p* = 0.001).

### Effect of Assortative Mating on Number of Fledglings

Competition, male, or female aggression did not influence the number of nestlings fledged (*male aggression: df = *1, *F* = 0.13, *p* = 0.72; *female aggression: df = *1, *F* = 1.24, *p* = 0.28; *competition: df = *1, *F* = 0.65, *p = *0.80). Further, we found no significant interactions between any independent variables (all p>0.3).

### Effect of Assortative Mating on Fledgling Size

Brood size (*df = *23.25, *F = *0.35, *p* = 0.71), nestling sex (*df* = 90.34, *F* = 0.32, *p = *0.57), and hatch date (*df* = 22.19, *F* = 0.23, *p* = 0.64) did not contribute significantly to the model of fledgling mass and were therefore excluded from further analyses. We found a significant interaction between competition level*male aggression*female aggression on nestling mass (*df = *33.41, *F* = 15.37, *p*<0.001). Because of this interaction, we split the dataset by high and low competition sites. In areas of low interspecific competition, we found no significant interaction between male and female aggression on nestling mass (*df = *14.56, *F* <0.01, *p* = 0.94). Further, neither male nor female aggression significantly affected nestling mass (Male aggression: *df = *14.69, *F* = 0.11, *p = *0.75; Female aggression: *df* = 13.60, *F* = 0.11, *p* = 0.92). However, in areas of high interspecific competition, we found a significant interaction between male and female aggression on nestling mass (*df* = 13.91, *F = *41.22, *p*<0.001).

**Figure 1:**
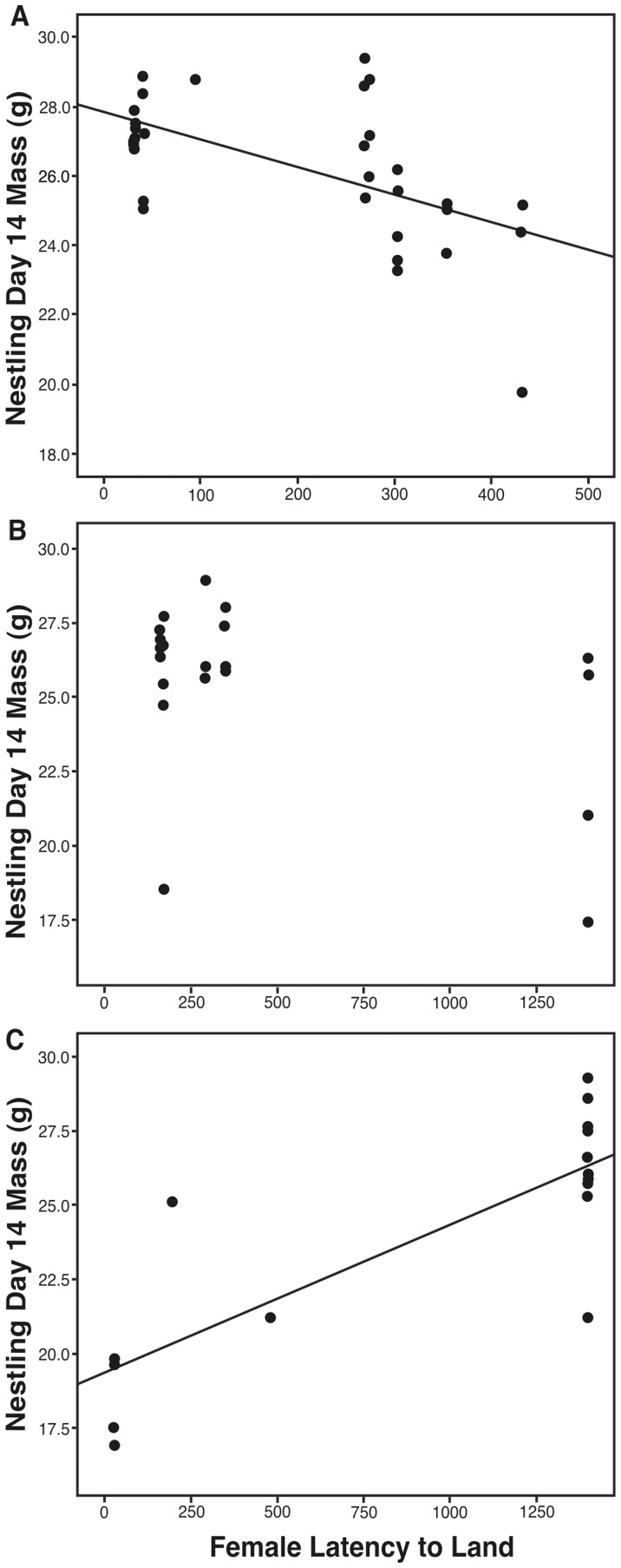
Effect of pair similarity on nestling quality (day 14 mass) in high competition sites. The graphs are split into groups for a) the most aggressive 3^rd^ of males, b) the middle 3^rd^ of males, and c) the least aggressive 3^rd^ of males. Aggression is measured as the latency to land on a simulated territorial intruder’s cage.

Because of the significant interaction between male and female behavior on nestling mass in the high competition dataset, we categorized male aggression into three groups: 1) most aggressive third of males, 2) middle third of males, and 3) least aggressive third of males [8]. We then ran separate models for each category of male behavior. Pairs that were mated assortatively on the extremes for aggression produced the heaviest nestlings (Fig. 1). When mated to highly aggressive males, female aggression was significantly positively related to nestling quality (*df = *6.63, *F = *8.30, *p = *0.03; Fig. 1a). When mated to males that were categorized as mid-level aggressive, female aggression did not significantly affect nestling quality (*df* = 3.50, *F = *4.25, *p* = 0.12; Fig. 1b). However, when mated to low-aggression males, female aggression was significantly negatively related to nestling quality (*df = *3.94, *F* = 10.51, *p* = 0.03; Fig. 1c). These data demonstrate that in high competition areas of the field site, pairs that show similar and the most extreme levels of aggression rear the heaviest offspring.

Overall, bluebird pairs in areas of low interspecific competition reared heavier nestlings compared to bluebird pairs that nested in areas of high interspecific competition (*df = *133, *t = *2.71, *p* = 0.008).

## Discussion

**Figure 2:**
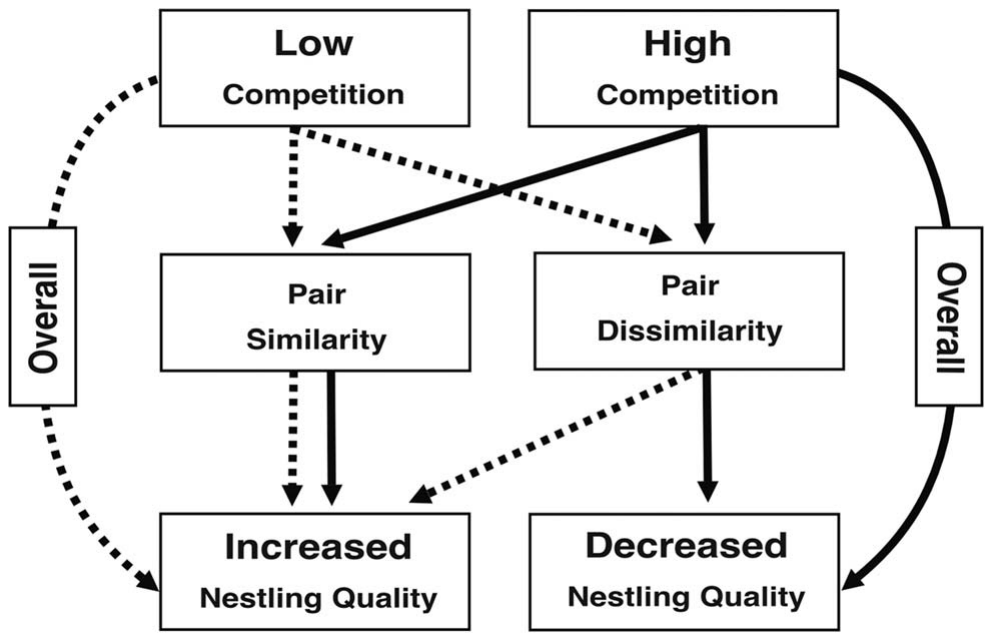
Overview of the effect of assortative mating on nestling quality. Solid lines represent high competition and dashed lines represent low competition environments. In either environment, pairs can either behave similarly or not. Our results suggest that nestling quality is higher in low compared to high competition sites, regardless of the degree of assortative mating. Pair similarity does not have a large effect on nestling quality in low competition sites, but in high competition sites, pair similarity significantly affects nestling quality. Pair similarity in high competition sites increases nestling quality and, conversely, pair dissimilarity decreases nestling quality.

Eastern bluebirds and tree swallows compete fiercely for nesting sites in some areas of our field site while, in other areas, competition is rare. Bluebird nestlings appear to suffer from competitive interactions with tree swallows because, at high-competition sites, nestlings are smaller at fledging age. When facing competition with tree swallows, pair similarity in aggression appears to strongly influence pair reproductive success; pairs that displayed similar and extreme responses to STIs fledged heavier offspring (see also Fig. 2 for a schematic overview). However, the total number of offspring fledged was not affected by parental behavior. This may be because we found little variation in brood sizes; 92% of pairs had broods of 3, 4, or 5 nestlings. Moreover, ‘brood reduction’, or the death of some nestlings within a brood, was rare (9.3% of broods). We also found evidence of assortative mating in all areas of the field site. It may be that the fitness benefits for assortative mating in the face of strong competition leads to the persistence of assortative behavior throughout the population.

Our data show fitness benefits for assortative pairs in high competition environments. This may be especially important in cavity-nesting species where defending a nesting cavity is extremely important and both sexes participate in nest defense. Great tit parents with similar personality types also produce higher quality young [9]. This relationship may occur because, when two highly aggressive birds mate, they are able to defend higher quality territories [9]; indeed, aggressive individuals have been shown to be more successful at securing high-quality nesting sites in a number of avian species [20], [50]–[53]. At the other end of the spectrum, pairs of great tits comprised of two non-aggressive individuals forage more efficiently even in lower quality habitats [14]. Our data, however, differ from those of Both et al. [9] in that the advantage of assortative mating was only obvious when bluebirds experienced high levels of interspecific competition with tree swallows. Because we conducted this study on populations using artificial nest boxes, our results are likely conservative. In populations where breeding birds occupy natural nesting cavities, interspecific competition is more intense than in box nesting populations [54]. Our study may provide insight into how assortative mating became widespread in bluebirds and similar trends may be expected to occur in other cavity nesting species as well.

An alternative explanation for the relationship between assortative mating and offspring quality is that parents with similar personalities may be better at coordinating parental care duties. Coordination of parental care behavior is important for success in birds that exhibit biparental care [55]. Spoon et al. [56] found that pairs of cockatiels (*Nymphicus hollandicus*) that behaved similarly coordinated incubation more efficiently leading to higher reproductive success. If bluebird pairs that behave similarly are better able to coordinate nestling provisioning, this may be particularly adaptive in a high competition environment where parents might need to invest more energy into defending the territory. Indeed, Meek and Robertson [57] found that in locations where male bluebirds spent more time defending the nest against tree swallows, they were less diligent in guarding their fertile mates. Trade-offs in energy investment may have a similar effect on parental provisioning rates. Perhaps when tree swallows harass bluebirds, a coordinated aggressive response by bluebird pairs allows them to spend less time defending the nest and more time provisioning young. A study of how competition influences the coordination of parental care and, in turn, how parental coordination influences offspring fitness would be helpful.

Many species mate assortatively for personality traits including great tits [58], zebra finches, *Taeniopygia guttata* [12], Stellar’s jays, *Cyanocitta stelleri* [59], bridge spiders, *Larinioides sclopetarius* [60], dumpling squids, *Euprymna tasmanica* [61], convict cichlids, *Cichlasom nigrofasciatum* [62], and humans [reviewed in 38], while white-throated sparrows, *Zonotrichia albicollis,* tend to mate disassortatively for aggression [63]. It may be that assortative mating for personality plays a role in sexual selection; however, it is difficult to know if the behavior of the individual changes after mating or if behavior is consistent and personality is an important criteria for mate choice. In this study, we measured territorial aggression, which may be a component of personality, but individuals in a pair that behave similarly may be simply responding to one another. In a captive setting where paired males and females were tested separately for boldness and exploratory behaviors (and where territorial aggression is not possible to measure), paired individuals did not display similar exploratory behaviors (Morris & Siefferman unpub. data). However, our study seeks to understand the consequences of individual behavior in an ecological setting. The way individuals behave in the presence of mates accurately reflects their behavior in natural conditions during the breeding season.

In this study, we demonstrated that female eastern bluebirds exhibit consistent territorial aggression while males do not. This sex difference in the consistency of aggressive response is similar to findings in an Oklahoma population of bluebirds [23]. These data suggest that if individuals are adjusting their behavior to match that of their mate, males might be adjusting more than females. However, this explanation is speculative because we do not know the behavior of the bluebirds before mate selection occurs. Nonetheless, there is widespread evidence for the benefits of assortative behavior suggesting it is an important component of fitness in many species [38].

One important limitation of our dataset, however, is that our proxy of fitness is limited to what can be measured at fledging (number and size of offspring). We do not yet understand how assortative mating for territorial aggression or how an individual’s level of aggression influences survivorship during the adult or juvenile stages. In other species, the benefits of particular personality traits lead to tradeoffs between survival and reproductive success [1], [10]. Ultimately, such trade-offs may maintain behavioral variation within a population.

The results we documented at the front of a natural range expansion demonstrate how new selection pressure can influence the fitness consequences of assortative mating. Tree swallows are extending their range southward [64] and have been in the NC study area <40 years [65] and from the perspective of bluebirds, are a highly aggressive invasive species. Invasive species are often more aggressive than native species [66], [67] and that could determine the success of invasions. One recent example is the endangered gouldian finch, *Erythrura gouldiae*, which is being out-competed by the more aggressive long-tailed finch, *Poephila acuticauda* [66]. As more invasive species are being introduced throughout the world, it is important to understand how aggression and other components of personality are affected and/or affect the outcome of competitive interactions. Animal personality has been largely overlooked in invasive species research, to date, despite its importance [7]. Our data shed light on how interspecific competition with aggressive invasive species exerts selection pressure on a less-aggressive, resident species and have far-reaching application toward understanding how behavior affects the vulnerability of species to invasions.
